# Behavioral trade‐offs and multitasking by elk in relation to predation risk from Mexican gray wolves

**DOI:** 10.1002/ece3.11383

**Published:** 2024-05-26

**Authors:** Zachary J. Farley, Cara J. Thompson, Scott T. Boyle, Nicole M. Tatman, James W. Cain

**Affiliations:** ^1^ Department of Fish Wildlife and Conservation Ecology New Mexico State University Las Cruces New Mexico USA; ^2^ New Mexico Department of Game and Fish Santa Fe New Mexico USA; ^3^ U.S. Geological Survey New Mexico Cooperative Fish and Wildlife Research Unit, Department of Fish Wildlife and Conservation Ecology New Mexico State University Las Cruces New Mexico USA

**Keywords:** antipredator behavior, foraging, multitasking, predation risk, predator–prey, vigilance

## Abstract

Predator non‐consumptive effects (NCE) can alter prey foraging time and habitat use, potentially reducing fitness. Prey can mitigate NCEs by increasing vigilance, chewing‐vigilance synchronization, and spatiotemporal avoidance of predators. We quantified the relationship between Mexican wolf (*Canis lupus baileyi*) predation risk and elk (*Cervus canadensis*) behavior. We conducted behavioral observations on adult female elk and developed predation risk indices using GPS collar data from Mexican wolves, locations of elk killed by wolves, and landscape covariates. We compared a priori models to determine the best predictors of adult female behavior and multitasking. Metrics that quantified both spatial and temporal predation risk were the most predictive. Vigilance was positively associated with increased predation risk. The effect of predation risk on foraging and resting differed across diurnal periods. During midday when wolf activity was lower, the probability of foraging increased while resting decreased in high‐risk areas. During crepuscular periods when elk and wolves were most active, increased predation risk was associated with increased vigilance and slight decreases in foraging. Our results suggest elk are temporally avoiding predation risk from Mexican wolves by trading resting for foraging, a trade‐off often not evaluated in behavioral studies. Probability of multitasking depended on canopy openness and an interaction between maternal period and predation risk; multitasking decreased prior to parturition and increased post parturition in high‐risk areas. Openness was inversely related to multitasking. These results suggest adult female elk are altering the type of vigilance used depending on resource availability/quality, current energetic needs, and predation risk. Our results highlight potentially important, but often‐excluded behaviors and trade‐offs prey species may use to reduce the indirect effects of predation and contribute additional context to our understanding of predator–prey dynamics.

## Graphical abstract

We quantified the effects of the Mexican wolf (*Canis lupus baileyi*) predation risk on elk (*Cervus canadensis*) behavior in the southwestern United States. Metrics that quantified both spatial and temporal predation risk were top predictors in both datasets. During midday when wolf activity was relatively low, the probability of foraging increased while resting decreased, in areas with high spatial predation risk. During crepuscular periods when elk and wolves were most active, increased predation risk was associated with increased vigilance and slight decreases in foraging. The probability of multitasking increased with predation risk, suggesting that adult female elk may be offsetting the non‐consumptive effects of risk on feeding time.

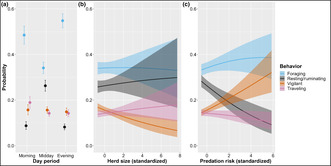

## 1 INTRODUCTION

The non‐consumptive effects (NCE) of predators can affect prey at multiple temporal and spatial scales (Eacker et al., 2016; Kittle et al., 2008; Lima & Dill, 1990; Middleton, Kauffman, McWhirter, Jimenez, et al., 2013). Some indirect effects of predation risk including changes in prey behavior and increased stress have the potential to reduce maternal nutritional condition and consequently, reduce birth rates and neonate survival (Barten et al., 2001; Christianson & Creel, 2010; Creel et al., 2007, 2013; Creel & Winnie, 2005; DeWitt et al., 2019; but see Middleton, Kauffman, McWhirter, Jimenez, et al., 2013; White et al., 2011). Yet, the indirect effects of predation risk are difficult to assess (Frair et al., 2005; Palmer et al., 2021; Proffitt et al., 2009) due to plasticity in the use of antipredator behaviors (Kohl et al., 2018; Lima & Dill, 1990; Proaktor et al., 2008) and behavioral trade‐offs used by prey which depend on various biological, ecological, environmental, social, and individual factors (Abernathy et al., 2022; Olsen et al., 2019; Proffitt et al., 2009). Prey likely utilize information (i.e., sensory, previous experience, social learning) about predators (e.g., space use, intensity of use, activity patterns, and landscape characteristics associated with risk) to assess risk. Prey can then alter their behavior to reduce their risk of predation to increase their likelihood of survival and potential fitness, adding to the difficulty of untangling these indirect effects.

The indirect effects of predators on prey are often studied by systematically observing the behavior of free‐ranging wild populations from afar due to the difficulty, cost, and invasiveness of obtaining long‐term data on individual nutritional condition. Given the links between nutritional condition, fitness, and population vital rates, studies often focus on the trade‐off between prey's ability to detect and ideally avoid predation (i.e., vigilance) with the need to acquire nutrition (i.e., foraging). Conversely, few studies have investigated the use of non‐foraging behaviors (e.g., traveling, resting) that prey may utilize as alternative trade‐offs or ways to mitigate the indirect effects of predators on foraging time.

According to the risk allocation hypothesis (RAH), prey can use different behaviors and behavioral trade‐offs to avoid predation depending on their age, sex, reproductive status, and motivational state (Fortin, Boyce, & Merrill, 2004; Kohl et al., 2018; Lima & Bednekoff, 1999; Proffitt et al., 2009; Valeix et al., 2009). One such behavior, vigilance, can reduce foraging time and intake rates (Fortin, Boyce, Merrill, & Fryxell, 2004; Illius & Fitzgibbon, 1994; Wolff & Horn, 2003). To mitigate the cost of increased vigilance, ungulates can synchronize their chewing with vigilance (i.e., multitasking; Fortin, Boyce, & Merrill, 2004; Fortin, Boyce, Merrill, & Fryxell, 2004; Illius & Fitzgibbon, 1994; Robinson & Merrill, 2013; Yiu et al., 2021). Vigilance while not chewing (intense vigilance) is considered a “stronger” form of vigilance due to it interrupting either foraging (Fortin, Boyce, & Merrill, 2004; Illius & Fitzgibbon, 1994) or digestion, as well as the reduction in noise and head movement allowing for better predator detection (Blanchard & Fritz, 2007). Thus, prey should adjust the use of intense and multitasking vigilance depending on various external factors such as predation risk and resource quantity/quality (Yiu et al., 2021). Additionally, ungulates can move to lower risk areas (i.e., spatial avoidance) and/or use high‐risk areas at low‐risk times (i.e., temporal avoidance; Kohl et al., 2018; Mao et al., 2005; Roberts et al., 2017). This plasticity can mitigate the indirect effects of predators by reducing the need for increased vigilance and/or allowing preferred foraging areas to be used without increased risk of direct predation. Finally, the motivational state of prey can influence drivers of and constraints on behavior. Foraging ungulates must balance the need to acquire food efficiently and simultaneously mitigate predation risk, whereas between foraging bouts, satiated animals should be primarily motivated to find safe places for resting and rumination. Ungulate prey typically have foraging‐rest/rumination cycles that follow a diel pattern with peaks in foraging during morning and evening crepuscular periods. Predators also have distinct activity patterns across the diel cycle, with peaks in hunting activity corresponding to diel periods most advantageous to their hunting style and prey accessibility (e.g., stalking versus coursing predators; Kohl et al., 2019). Quantifying the behavioral changes made by prey in relation to various ecological, biological, and environmental factors will increase our understanding of predator–prey dynamics in relation to risk allocation.

According to the reproductive strategy hypothesis (RSH), mothers, a demographic group that faces additional constraints on their fitness due to maternal investment and care (Hamel et al., 2010; Pekins et al., 1998; Robbins & Robbins, 1979), will balance foraging with predation risk for not only themselves but also their offspring (Main, 2008; Mysterud, 2000). On one hand, investment into healthier/heavier offspring and predator detection/avoidance increases offspring survival and hence the mother's fitness (Gaillard & Yoccoz, 2003; Griffin et al., 2011; Morano et al., 2013; Stearns, 1992; Thorne et al., 1976). On the other hand, maternal investment and care (e.g., lactation; Gittleman & Thompson, 1988) can be costly, requiring a trade‐off between long‐term adult survival, lifetime reproductive success, and short‐term offspring survival (Morano et al., 2013; Thorne et al., 1976). The trade‐off between long‐term adult survival and recruitment of young should promote the evolution of strategies that increase offspring survival while decreasing maternal costs. For example, when neonate vulnerability is high, ungulate mothers will often select safer habitats, even at the expense of access to improved foraging conditions, and reduce movement to remain near offspring to provide increased access to milk and predator detection via increased vigilance (Carl & Robbins, 1988; Costelloe & Rubenstein, 2015; Fitzgibbon, 1990; Lent, 1974; Vore & Schmidt, 2001). During periods of restricted mobility, mothers likely need to adjust their foraging strategy to mitigate negative consequences on their own nutritional condition. Once young are more mobile and better able to avoid predators, the movement constraints for mothers are relaxed, allowing for more adaptive foraging movements and reduced vigilance; this typically coincides with a decrease in suckling (Bongi et al., 2008; Costelloe & Rubenstein, 2015; Vore & Schmidt, 2001). This plasticity in foraging and vigilance behavior has been shown in multiple ungulate species in relation to their perceived predation risk (Bongi et al., 2008; Viejou et al., 2018; Winnie & Creel, 2007).

Mexican gray wolves (*Canis lupus baileyi*; hereafter Mexican wolves) were extirpated from the southwestern United States by the 1970s and reintroduction began in 1998. They are currently a federally listed, endangered species and as of 2022, the minimum Mexican wolf population in Arizona and New Mexico was 241 individuals (USFWS, 2023). Current management for Mexican gray wolf recovery is to increase the population by at least 32% to meet the goals in the current recovery plan (USFWS, 2017). In Arizona and New Mexico, elk (*Cervus canadensis*) are the primary prey of Mexican wolves (Reed et al., 2006; Smith et al., 2023). Elk are a socially, economically, and ecologically important species that generate millions of dollars annually via hunters and wildlife viewers which funds state management agencies and local economies (Southwick Associates, 2014). In addition to Mexican wolves, adult elk also compose a large portion of the diet of mountain lions (*Puma concolor*), while black bears (*Ursus americanus*), coyotes (*Canis latrans*), and bobcats (*Lynx rufus*) regularly prey on elk calves. This system, in which a primary predator population is reestablishing after it was absent for >20 years and now exhibits varying densities across the landscape, provides a unique opportunity to quantify their NCEs on their primary prey. In addition, understanding these effects is important for making informed management and conservation decisions as well as furthering our knowledge on how prey may adapt their behavior to mitigate the indirect effects of predators, an important aspect of predator–prey dynamics.

Our primary goal was to determine how variation in Mexican wolf predation risk is related to the behavior of adult female elk and if elk utilize mitigating behaviors to reduce the effects of predation risk (e.g., spatial/temporal avoidance or multitasking). We expected adult female elk to take advantage of temporal and spatial changes in risk to reduce the indirect effects of predators (Kohl et al., 2018, 2019) as outlined in the RAH. However, when elk do not temporally or spatially avoid predation risk, we expected increased use of vigilance along with a potential reduction in time allocated to other behaviors (e.g., foraging). Similarly, we expected elk to increase their use of multitasking (vigilant while chewing) to mitigate the cost of increased vigilance in high‐risk areas/times but their use of multitasking vs intense vigilance would also depend on the reproductive state (i.e., pre or post parturition). In line with the RSH, we expected adult female elk nutritional needs related to gestation/lactation and calf susceptibility to predation to be important predictors of behavior. We predicted vigilance to be lowest and foraging to be highest prior to parturition, followed by a reduction in foraging and an increase in vigilance when neonates are most vulnerable to predation (Eacker et al., 2016; Griffin et al., 2011). Following the dilution and detection (Dehn, 1990) hypotheses, we expected herd size to be related to adult female behavior with individual vigilance decreasing as herd size increases. We expected that individual vigilance would decrease with an increase in the proportion of females due to the reduced number of calves in the herd. Lastly, because landscape characteristics related to forage availability and predator detection are associated with elk behavior, we assessed if adult female elk behavior is best predicted by the inclusion of characteristics associated with the structure/composition and distance to various vegetation features that might enhance predator avoidance by prey or hunting success by predators. We expected that vigilance would increase closer to increased canopy cover due to a reduction in the effectiveness of vigilance and increased escape impediments. Lastly, we expected the ratio of intense to multitasking vigilance to alter according to resource abundance/quality (i.e., spare time).

## 2 STUDY AREA

**FIGURE 1:**
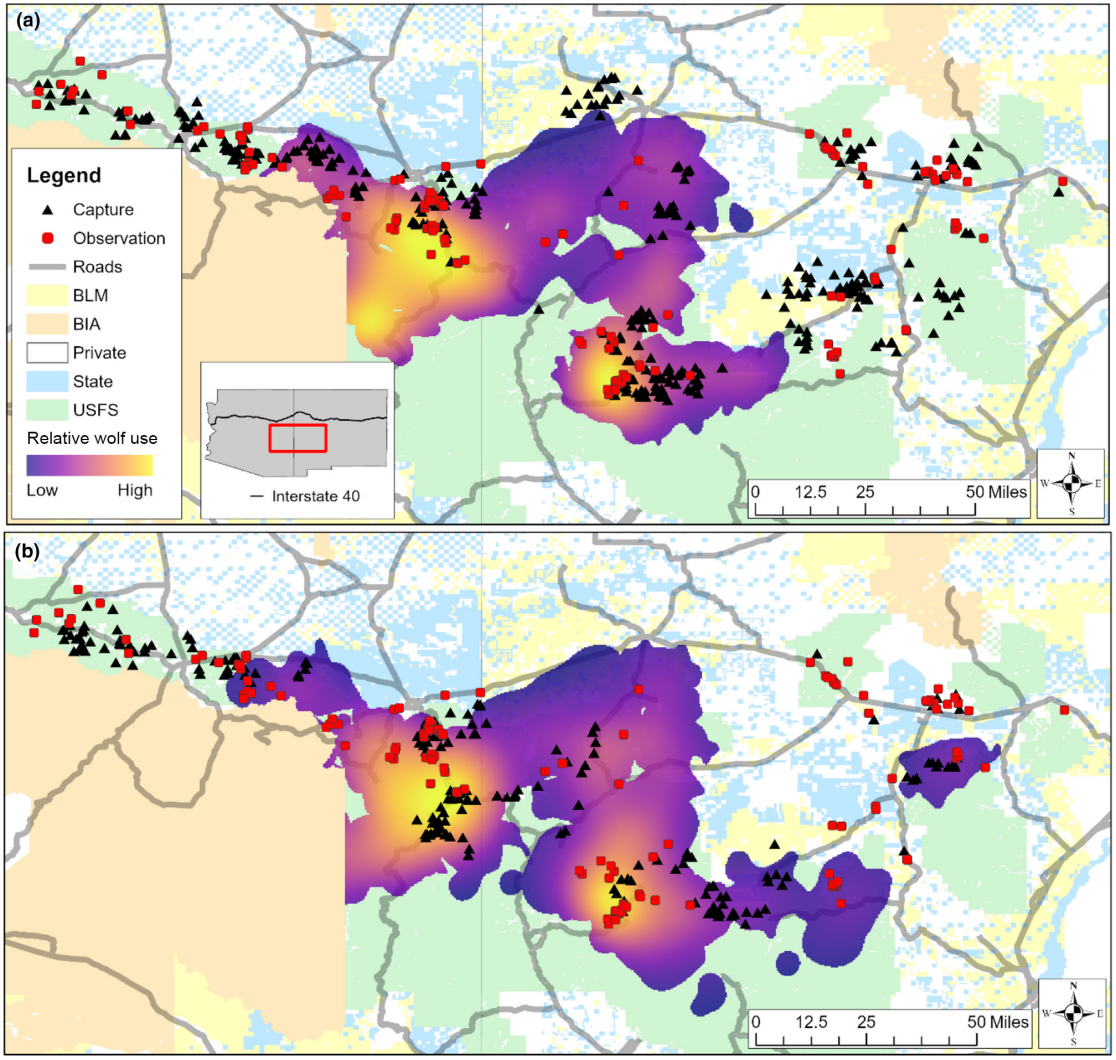
Study area including land ownership, roads, capture/observation locations, and yearly population‐level Mexican wolf utilization distributions overlain on pack boundaries for 2019 (a) and 2020 (b). Only packs known to exist ≥6 months are included.

The Mexican Wolf Experimental Population Area in eastern Arizona and western New Mexico, USA, is delineated by Interstate 40 to the north and Mexico to the south. Land ownership in our 25,000 km^2^ study area is primarily national forests (i.e., Apache‐Sitgreaves [A‐SNF], Gila, and Cibola National Forests) interspersed with private, state trust, and Bureau of Land Management lands (Figure 1). Tribal lands including the White Mountain and San Carlos Apache border the A‐SNF to the south and west in Arizona. Wolf density in areas occupied by Mexican wolves varies throughout the study area, ranging from areas with no known occupation by established packs to areas with consistent occupation with multiple adjacent packs (Figure 1).

Topography varies from moderately steep canyons and sandy washes within lowland rolling hills to rugged slopes, mesas, cliffs, and deep canyons at high elevations; elevation ranges from 1700 to 3477 m (USFWS, 1996). Water sources include man‐made tanks as well as natural springs, lakes, and streams with the Gila and San Francisco rivers constituting the major drainages (USFWS, 1996).

Lower elevations (≤1975 m) are characterized by warm summer (June–August) daily high/low temperatures (30.2°C/11.9°C), while higher elevations (≥2684 m) are cooler (22.5°C/7.7°C; PRISM Climate Group, 2021). Approximately half of the annual precipitation occurs during the summer monsoon season (June–September). The average total precipitation during the monsoon season is 33.6 cm ± 7.9 cm (SD) and 19.3 cm ± 5.0 cm (SD) in high‐elevation areas and low‐elevation areas, respectively (PRISM Climate Group, 2021). Precipitation predominantly occurs as snowfall in the high‐elevation areas from December to March with an average of 193.2 cm ± 105.3 cm (SD; Western Regional Climate Center, 2019).

Vegetation communities transition from desert scrub and plains at lower elevations to pinyon‐juniper (*Pinus edulis‐Juniperus* spp.) woodland and ponderosa pine (*Pinus ponderosa*) forests. Higher elevations are mixed‐conifer and spruce‐fir forests interspersed with montane grasslands; common species include Douglas‐fir (*Pseudotsuga menziesii*), white fir (*Abies concolor*), blue spruce (*Picea pungens*), aspen (*Populus tremuloides*), Arizona fescue (*Festuca arizonica*), pine dropseed (*Blepharoneuron tricholepis*), and mountain muhly (*Muhlenbergia montana*; Brown, 1982). A variety of understory shrubs, grasses, and forbs are present as well (USFWS, 1996). Both natural and human‐caused fires are interspersed throughout the area including the 120,534 ha. Whitewater‐Baldy Complex Fire in New Mexico in 2012 and the 217,721 ha. Wallow Fire in the A‐SNF in 2011, both of which predominantly occurred in high wolf density areas. Cattle grazing is common, with many areas stocked year‐round. Both consumptive (e.g., hunting, fishing) and non‐consumptive (e.g., hiking, camping, horseback riding) public recreation occurs throughout the study area.

Ungulates in the area include elk, Coues white‐tailed deer (*Odocoileus virginianus couesi*), mule deer (*Odocoileus hemionus*), pronghorn (*Antilocapra americana*), Rocky Mountain bighorn sheep (*Ovis canadensis canadensis*), and collared peccary (*Pecari tajacu*) (USFWS, 1996). The Mexican wolf, black bear, mountain lion, coyote, bobcat, and gray fox (*Urocyon cinereoargenteus*) are common predators (USFWS, 1996).

## 3 METHODS

### 3.1 Animal capture

We captured 675 adult female elk using a net gun fired from a helicopter, corral traps, clover traps, or ground darting from January through early April in 2019 and 2020 (Figure 1); we captured additional elk via ground darting in August of each year. Elk captured via net gun were physically restrained and all others were immobilized with 2 cc of premixed butorphanol tartrate (27.3 mg/mL), azaperone (9.1 mg/mL), and medetomidine (10.9 mg/mL; BAM). After a minimum of 20 min, we administered 4 cc of atipamezole (25 mg/mL) and 0.5 cc of naltrexone (Wolfe et al., 2014). Captures were randomly stratified by relative high and low wolf density areas to evaluate the influence of varying Mexican wolf predation risk (hereafter predation risk) on elk behavior. We fitted all elk with a unique ear tag combination and a GPS‐Iridium collar (ATS, model G5‐2D, Isanti, MN) programmed with either a 2‐ or 4‐h GPS fix interval. GPS data were transmitted every 4 days except during parturition (mid‐May–mid‐June) when data were transmitted twice a day. Pregnancy was determined via pregnancy‐specific protein B (PSPB) assays from blood collected during capture (Drew et al., 2001).

In May–June of 2019 and 2020, we captured 419 neonate elk either opportunistically, via helicopter, or by using GPS collar location data from adult female elk to derive movement‐based metrics that infer parturition (DeMars et al., 2013). We captured a subset of calves from collared adult females each year. We estimated parturition dates for captured calves following Eacker (2015). All animal capture and handling procedures were approved by New Mexico State University Institutional Animal Care and Use Committee (IACUC protocol #2018‐019).

### 3.2 Behavioral observations

To conduct behavioral observations, we located elk opportunistically or using VHF telemetry from January 1st through the 1st week of September in 2019 and 2020 to avoid any potential direct anthropogenic influence due to human hunting activities (Proffitt et al., 2009). We observed elk via binoculars (10 × 42) or spotting scopes (20–60 × 85) at distances ranging from 30 to 4500 m (mean 479.3 m ± 455.5 [SD]) between morning and evening civil twilight. We used instantaneous sampling and recorded the behavior of individual elk once every minute, on the minute, beginning at the start of the observation (Roberts et al., 2017). We observed collared individuals when possible, otherwise, we selected focal individuals using a randomly generated compass bearing and number indicating the direction/number of individuals away from the center point of the herd. If multiple animals with collars were present in the same herd, we selected the individual with the fewest observations. To avoid non‐independent samples, we observed no more than four uncollared individuals during a session and we did not observe collared individuals more than once in the same day. We classified behaviors as: (1) foraging (browsing, grazing, chewing with head up [but not vigilant] or down or moving slowly with head below shoulder level); (2) vigilant (immobile with head above or at shoulder height while standing or lying on the ground with a fixed and alert gaze); (3) resting/ruminating (lying on the ground or standing not vigilant or exhibiting any other behavior); (4) traveling (walking or running with head at or above shoulder level); (5) nursing; (6) drinking; and (7) other (aggression, grooming, or mating). When individuals were vigilant, we also recorded if they were chewing, including while bedded, to quantify multitasking. If an individual's behavior could not be accurately determined because it was temporarily obscured from view, we recorded it as unknown and removed it from the analysis. If an elk showed signs of being disturbed due to anthropogenic causes (e.g., passing vehicles, hikers, or observer influence), their behavior was recorded as such and removed from the analysis.

We also recorded time, date, location of the observation, dominant vegetation type, herd size, and herd composition. The mean herd size across all observations was 27 ± 33 (SD). We recorded the location of observations either using GPS collar data, when available, or a handheld GPS unit, rangefinder, and compass to offset the herd location relative to the observer's position. We defined demographic groups as: calves—individuals <1 year old, cows—females >1 year old, bulls—males with antlers with at least one brow tine, and spikes—males with antlers without brow tines (Liley & Creel, 2008).

### 3.3 Maternal periods

We classified observations into one of four periods based on maternal investment/care and calf mobility (i.e., potential to escape predators; Eacker et al., 2016; Griffin et al., 2011; Morano et al., 2013). The pre‐parturition period (PPP; duration ca. 50 days) began on April 1st and continued until the day before parturition. For observations on adult females whose calves we captured, we used our estimated parturition date to determine the end of the PPP. For adult females from which we did not capture calves, we used the mean Julian day for all estimated parturition dates of all captured calves (152 Julian day), then subtracted three standard deviations (17.4 days), thus reducing the possibility of observing a female post parturition unknowingly. The limited mobility period (LMP; ca. 45 days) began the day after the PPP ended (Eacker et al., 2016). During the LMP, calves are most vulnerable to predation largely due to their inability to escape predators (Eacker et al., 2016; Griffin et al., 2011; Tatman et al., 2018). The social mobility period (SMP; ca. 60 days) began the day after the end of the LMP and ended approximately the first week of September. Generally, the probability of calf predation decreases throughout the SMP as calves become more mobile (Eacker et al., 2016; Griffin et al., 2011; Tatman et al., 2018). Winter period (WP) was from January 1st to March 31st for all observations (ca. 90 days). By winter, calves no longer rely on lactation for nutrition and are physically capable of avoiding predators similar to adults (Toweill & Thomas, 2002). We conducted 709 focal observations (PPP *n* = 103, LMP *n* = 182, SMP *n* = 345, WP *n* = 79) lasting between 10 and 40 min (19 ± 5 [SD]) from morning to evening civil twilight (morning *n* = 134, midday *n* = 338, evening *n* = 237). Predators were seen affecting elk behavior on 4 (0.45%) occasions across all observations.

### 3.4 Predation risk

The Mexican Wolf Interagency Field Team documented new packs and attempted to maintain at least one GPS collar in every known pack. Approximately 50%–60% of the Mexican wolf population was fitted with GPS collars, allowing for robust estimation of spatiotemporal predation risk metrics. How prey recognize spatial and temporal variation in predation risk is still uncertain (Kauffman et al., 2013; Moll, 2017; Suraci et al., 2022). Therefore, we used three primary predation risk metrics (predicted wolf presence, risky places, and risky places in relation to wolf use) and two secondary metrics of predation risk (openness and wolf activity) developed by Thompson (2022) to assess the NCEs of Mexican wolves on elk behavior (Appendix 1: Table A1).

### 3.5 Predicted wolf presence

Utilization distributions (UDs) quantify space use by animals and represent an approximation of predation risk by revealing spatial–temporal overlap between predators and prey, resulting in an increased likelihood of encounters (Hebblewhite & Merrill, 2007; Viejou et al., 2018; but see Suraci et al., 2022). We used GPS collar data from Mexican wolves in 2019 and 2020 to create UD rasters (380 × 450‐m resolution) from kernel density estimates at four spatial–temporal scales (pack‐season‐year [PackSY], Pack by year using the maximum pack count [PackYm], population‐season‐year [PopSY], population by year [PopY]; Thompson, 2022; Appendix 2). Population UDs pooled data across packs, resulting in a coarser landscape scale depiction of space use compared to pack UDs which were estimated separately for each pack. Yearly UDs did not account for seasonal variation in space use while season UDs did. To estimate the population level UDs, we used a single individual with the most data from each pack. To account for variation in risk due to wolf pack size, we multiplied each PackSY UD pixel value by the corresponding pack size during respective seasons (USFWS, unpublished data). For the PackYm UD, we used the maximum number of wolves in each pack as a conservative approach to estimating wolf presence on the landscape. Where separate pack UDs overlapped, the corresponding pixel values were summed.

Prey can recognize landscape attributes (i.e., indirect cues) associated with an increased risk of encounters with predators (Ditmer et al., 2018; Gaynor et al., 2019). To account for this understanding, we developed a resource selection function (RSF) using mixed effects logistic regression, utilizing GPS locations from collared Mexican wolves while accounting for season, as defined for UDs (Hebblewhite & Merrill, 2007; Mao et al., 2005; Appendix 2). We extracted (UD) and calculated (RSF) values for each observation based on its location and date. Because we currently do not know at which scales prey perceive the intensity of space use by predators, we extracted mean UD values from a circular buffer around each observation at three spatial scales: 200, 800, and 2000 m (Basille et al., 2015; Middleton, Kauffman, McWhirter, Jimenez, et al., 2013; Proffitt et al., 2009). We then took the product of the UD and RSF as our metric of predicted wolf presence (Hebblewhite & Merrill, 2007).

### 3.6 Risky places relative to wolf use

Characteristics of predation sites can be a strong indicator of risk for prey (Hebblewhite & Merrill, 2007, 2009; Kauffman et al., 2007). We located elk killed by Mexican wolves using mortality data from elk fitted with GPS collars and by investigating GPS clusters created by collared Mexican wolves (Appendix 3; Sand et al., 2005; Webb et al., 2008). Using mixed effects logistic regression, we incorporated landscape attributes at sites where elk (calf and adult female) were killed by Mexican wolves and random locations within respective pack territories to determine the best predictors of these kill sites (i.e., risky places [RP], Appendix 3; Kauffman et al., 2007, 2010). We calculated the RP value for each observation using the beta estimates of the predictors from the most supported model (Appendix 3). We then calculated the product of UD and RP values for each observation to create the metric of risky places relative to wolf use.

### 3.7 Wolf activity

Movement rates are an appropriate proxy for the activity of large mammals (Ensing et al., 2014). When temporal activity patterns of predators are predictable, prey may alter their antipredator behavior to take advantage of this consistency. For example, prey may reduce their vigilance during periods of low predator activity, thus reducing the costs of vigilance (Lima & Bednekoff, 1999). Following Kohl et al. (2018), we used generalized additive mixed models to account for the variation in seasonal movement rates (km/2‐h) of 27 Mexican wolves from 25 packs across diel cycles between 2017 and 2020 (Thompson, 2022). To account for the skewness of movement data and variation between individual wolves, we used a negative binomial distribution and a random intercept for wolf ID, respectively. We then calculated predicted Mexican wolf movement rates for each elk observation based on the time and season of the observation.

### 3.8 Openness

Mao et al. (2005) found that elk altered their use of open habitats after wolf recolonization by selecting less open areas in the summer and more open areas in the winter. Hebblewhite et al. (2005) reported that elk were far more likely to be encountered in grasslands than forests during winter but the odds, relative to encounter, of elk being killed by wolves were much higher in pine stands than grasslands. Kohl et al. (2018) found that elk avoided more open areas when wolf activity was high, but selected open areas when wolf activity was low. We used a metric for habitat openness using GIS data on tree canopy cover (NLCD, 2016). Across the study area, we summed the number of 30 × 30‐m pixels centered around a focal pixel categorized as open canopy cover (≤30% canopy cover) within a 500 × 500‐m moving window (Kohl et al., 2018; Mao et al., 2005). We extracted openness values for each observation from the derived openness raster based on its location.

### 3.9 Non‐risk factors affecting elk behavior

To account for diurnal changes in elk behavior we delineated three periods (morning, midday, and evening) relative to civil twilight using estimated movement rates from elk GPS collar data with the package *suncalc* in Program R (Ensing et al., 2014; R Core Team, 2022; Thieurmel & Elmarhraoui, 2019). Morning occurred from morning civil twilight to 2.5 h after. Evening encompassed evening civil twilight to 2 h prior. Midday was the diurnal period between morning and evening. We assigned observations to one of three diurnal periods based on its location, date, and time. We assigned observations that spanned two diurnal periods to the period during which most of the observation occurred. To evaluate if elk behavior was better predicted by condensing periods where activity is similar, we combined morning and evening periods when elk are commonly feeding.

In addition to the impact of diurnal period on elk behavior, we were also interested in how biological, landscape, and social covariates influenced individual behaviors. For example, herd size, proportion of females in a herd, cover, and distance to cover have been shown to influence elk behavior (Liley & Creel, 2008; Robinson & Merrill, 2013; Appendix 4: Table A6). We calculated cover using a combination of vegetation type, height, and coverage at 30‐m resolution into two categories: open and cover (LANDFIRE, 2020). Open areas included any vegetation type with percent coverage ≤16% and vegetation height ≤2 m and cover was defined as areas that had tree and shrub cover ≥17% and height >2 m. We estimated the distance to cover in program R (R Core Team, 2022). We calculated the proportion of females and herd size using the herd composition data collected during observations.

## 4 ANALYSIS

### 4.1 Elk activity budgets and antipredator responses

In our study we sought to (1) assess how variation in Mexican wolf predation risk is related to behavior of adult female elk, and (2) determine if elk make adjustments to their activity budgets in a manner that would mitigate the indirect effects of predation risk (e.g., spatial/temporal avoidance or multitasking). Therefore, we incorporated variables expected to influence elk behavior based on our hypotheses and peer‐reviewed literature (Appendix 4: Table A6) into 11 a priori models (Appendix 4: Table A7).

Prior to developing a priori models in line with our hypotheses, we evaluated all predictor variables for collinearity using the appropriate analysis based on data types (i.e., ANOVA or Kruskal–Wallis for continuous × categorical or Spearman's rank correlation coefficient [<0.7] for continuous × continuous). If any covariates were collinear, the most ecologically relevant was used. In situations when categorical and continuous variables of interest were not independent but ecologically important, we included their interaction term to evaluate their importance. If we found the interaction(s) to (1) not greatly affect the standard errors of the estimates, (2) not be significant via their credible intervals, and (3) not outperform (see below) their respective models without the addition of the interaction terms, then we retained more parsimonious models without the additional terms. We centered and scaled all continuous predictor variables prior to running models (Schielzeth, 2010). To increase model interpretability, we only include two‐way interactions. In top models, we compared diurnal period consisting of either two or three periods. We set the reference categories as: traveling for behavior, midday for diurnal period, open for cover, and limited mobility for maternal period. Due to limited sample size, we combined nursing and drinking with the category “other.”

Due to the compositional nature of the response variable (i.e., behavioral proportions summing to one), we used the alternative parameterization of Dirichlet regression with a logit link for each behavior category in a Bayesian framework (Douma & Weedon, 2019; Wang, 2021). When analyzing compositional data via Dirichlet regression, the likelihoods of proportions equal to either 0 or 1 cannot be calculated (Douma & Weedon, 2019; Martín‐Fernández et al., 2003), therefore, we transformed these proportions following Smithson and Verkuilen (2006). We evaluated the importance of two random variables: individual (i.e., repeated observations on the same individual) and herd (i.e., correlation of behavior between individuals within the same herd), to account for the hierarchical nature of the data (Harrison et al., 2018). Only the random variable for herd was influential and therefore used in all models.

### 4.2 Multitasking

We utilized generalized linear mixed effects logistic regression with nine a priori models to evaluate which variables best predicted multitasking (Appendix 4: Table A8). We included herd and individual as random effects in preliminary analyses and found their inclusion did not influence model estimates. To account for multiple instances of multitasking occurring within a single observation and unequal sample sizes between observations, we used a random effect for observation. We evaluated the effect of diurnal period on multitasking by comparing all a priori models with and without the diurnal period term.

### 4.3 Predation risk

To determine the most influential metric of predation risk on elk behavior, we compared three primary metrics (predicted wolf presence, risky places in relation to wolf use, and risky places) alone and with each of the secondary metrics (openness and wolf activity) separately, both additively and multiplicatively. To account for the potential spatiotemporal variation in the ability of elk to perceive risk, we evaluated the various UD spatial and temporal scales within the top models for each dataset.

Post hoc, for each respective dataset, we compared log transformed predation risk metrics to their non‐log counterparts in an attempt to reduce credible intervals. If interactions were detected with the a priori models, we further investigated these by creating additional models and comparing them to previous top performing models within respective datasets.

### 4.4 Bayesian model specification, diagnostics, and comparison

We used uniform priors for all beta estimates and weakly informative priors in the form of the Student's *t*‐distribution with degrees of freedom = 3, location = 0, and scale = 2.5 for the intercepts of the response variable (*c* = 5 for focal models or *c* = 2 for multitasking models), and random effect standard deviation estimation (Lemoine, 2019). We used the gamma log density distribution as the prior for estimating phi with shape = 0.01 and rate = 0.01 for all Dirichlet models. For group level effects, we used the standard normal log density distribution with location = 0 and scale = 1 as the prior. We ran models using either four chains, 4000 warmup iterations, and 4000 sampling iterations per chain or 16 chains, 1000 warm up iterations, and 1000 sampling iterations per chain both resulting in 16,000 warm up iterations and 16,000 sampling iterations with the *brms* package (Bürkner, 2017) in program R (R Core Team, 2022). Computational time necessitated the use of a high‐performance computer, allowing for greater parallelization resulting in a different number of chains between some models.

Model diagnostics included visually inspecting the MCMC chains mixing, autocorrelation plots, and posterior distribution shape. We checked for divergent iterations and saturated maximum tree depth. Additionally, we checked all R‐hat values were ≤1.01 and ensured posterior effective sample sizes were large (i.e., >10 times the number of chains and *n*_eff_/*N* > 0.5; Gelman et al., 2013).

For activity budgets and antipredator response models, when applicable, we first compared the various predation risk metrics within each a priori model structure via their respective estimated expected log pointwise predictive density (ELPD) derived from 10‐fold cross‐validation (Hooten & Hobbs, 2015). We then compared all a priori models using their respective best‐performing predation risk metric. For multitasking models, we used the same process but compared models via their estimated ELPD using Pareto smoothed importance‐sampling leave‐one‐out cross‐validation (PSIS‐loo‐cv; Vehtari et al., 2016). If models did not contain a significant predictor of interest (i.e., interaction if included), they were removed from the final comparison. For all datasets, models with an ELPD difference of <16 from the top ranked model were retained across the iterative comparison process. To aid in differentiating models with similar ELPD values and overlapping standard errors we calculated model weights using pseudo‐Bayesian model averaging with Bayesian bootstrap (Höge et al., 2020; Yao et al., 2018). If the top model was >4 ELPD from the next model (Sivula et al., 2020) or carried the majority of the calculated weight (Yao et al., 2018), we considered it the sole top model.

## 5 RESULTS

### 5.1 Elk activity budgets and antipredator responses

**FIGURE 2:**
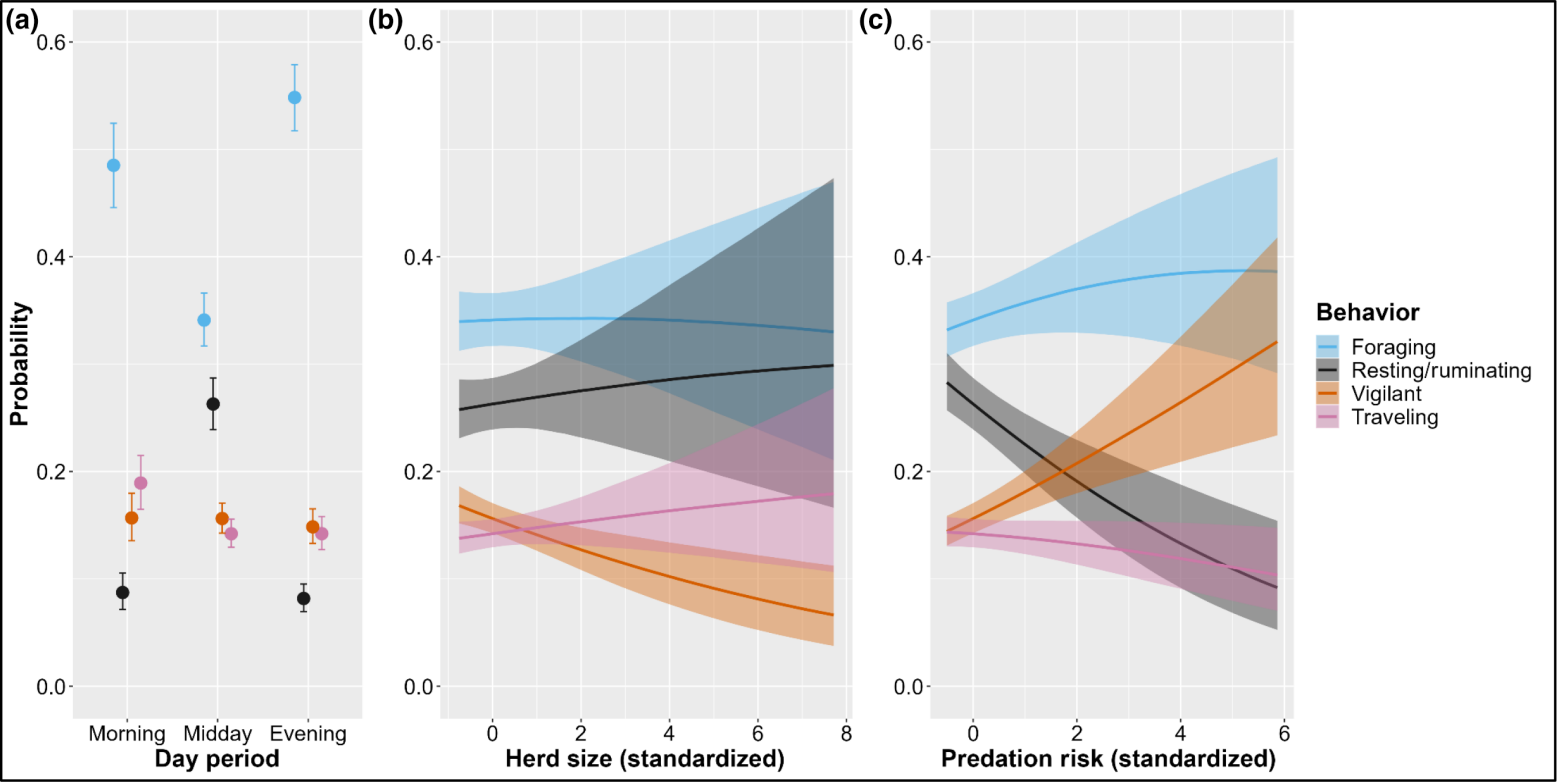
Influence of (a) diurnal period, (b) herd size, and (c) Mexican wolf predation risk on adult female elk behavior in west‐central New Mexico and east‐central Arizona 2019–2020. Parameters estimated from the most supported sampling model of activity budgets and antipredator responses data. Each panel shows the conditional effect of a predictor on the estimated probability of each behavior with 90% credible intervals. The behavior “other” is removed for clarity.

Behavior was best predicted by diurnal period with three periods, herd size, and predation risk (Table 1). The effects of the predictors in the top model were consistent across all models. The probability of foraging was 42.2% higher during the morning (0.446–0.524 [90% Credible Interval (CrI)]) and 60.7% higher during the evening (0.517–0.579 [90% CrI]) than during midday (0.317–0.366 [90% CrI]), whereas the probability of resting/ruminating was 202.3% higher during midday (0.239–0.287 [90% CrI]) than the morning (0.072–0.105 [90% CrI]) and 220.7% higher than during the evening (0.07–0.095 [90% CrI]; Figure 2a). The probability of traveling was greatest during the morning (0.165–0.215 [90% CrI]). Only vigilance was related to differences in herd size with the probability of an adult female being vigilant decreasing with increasing herd size (Figure 2b). Of the predation risk variables tested, predicted wolf presence using the PackYm UD at the 200‐m scale performed best (Table 1). As predation risk increased, the probability of foraging and vigilance also increased while resting/ruminating decreased (Figure 2c).

**TABLE 1:** The best‐performing models predicting adult female elk behavior from focal sampling in west‐central New Mexico and east‐central Arizona 2019–2020.

Model^a^ ^,^ ^b^ ^,^ ^c^	ELPD diff	SE diff	ELPD kfold	SE ELPD kfold	P kfold	SE p kfold	PBMA BB Wts
Diurnal Period + Herd Size + PackYm200‐RSF	0	0	7877.94	121.62	343.03	14.25	0.53
Diurnal Period + Proportion Cows + PopSY2000‐RSF	−5.93	12.27	7872.02	121.32	337.88	14.57	0.24
Diurnal Period + Maternal Period + Distance to Cover + PopY200‐RSF	−5.96	11.36	7871.99	120.65	358.26	15.44	0.22
Diurnal Period + Maternal Period * PopSY200‐RSF + Distance to Cover * PopSY200‐RSF	−19.28	12.04	7858.67	121.22	379.05	15.92	0.01
Diurnal Period + Herd Size	−32.25	10.57	7845.70	121.16	354.09	15.40	0.0
Diurnal Period + Proportion Cows	−33.18	10.74	7844.77	121.09	352.81	15.12	0.0
Null	−82.72	18.19	7795.22	121.5	350.57	14.21	0.0

*Note*: Column definitions: ELPD diff = difference in ELPD value between model and top model. SE diff = difference in standard error of ELPD between model and top model. ELPD kfold = ELPD value from 10‐fold cross validation. SE ELPD kfold = standard error of the ELPD kfold value. P kfold = effective number of parameters. SE p kfold = standard error of p kfold value. PBMA BB Wts = pseudo‐Bayesian model averaging with Bayesian bootstrapping weights.

^a^ Within models all main effects of variables in interactions are included but not shown.

^b^ Predation risk indices were created prior to modeling. The first variable indicates the wolf UD used and what scale, in meters.

^c^ All models included a random term for herd.

**FIGURE 3:**
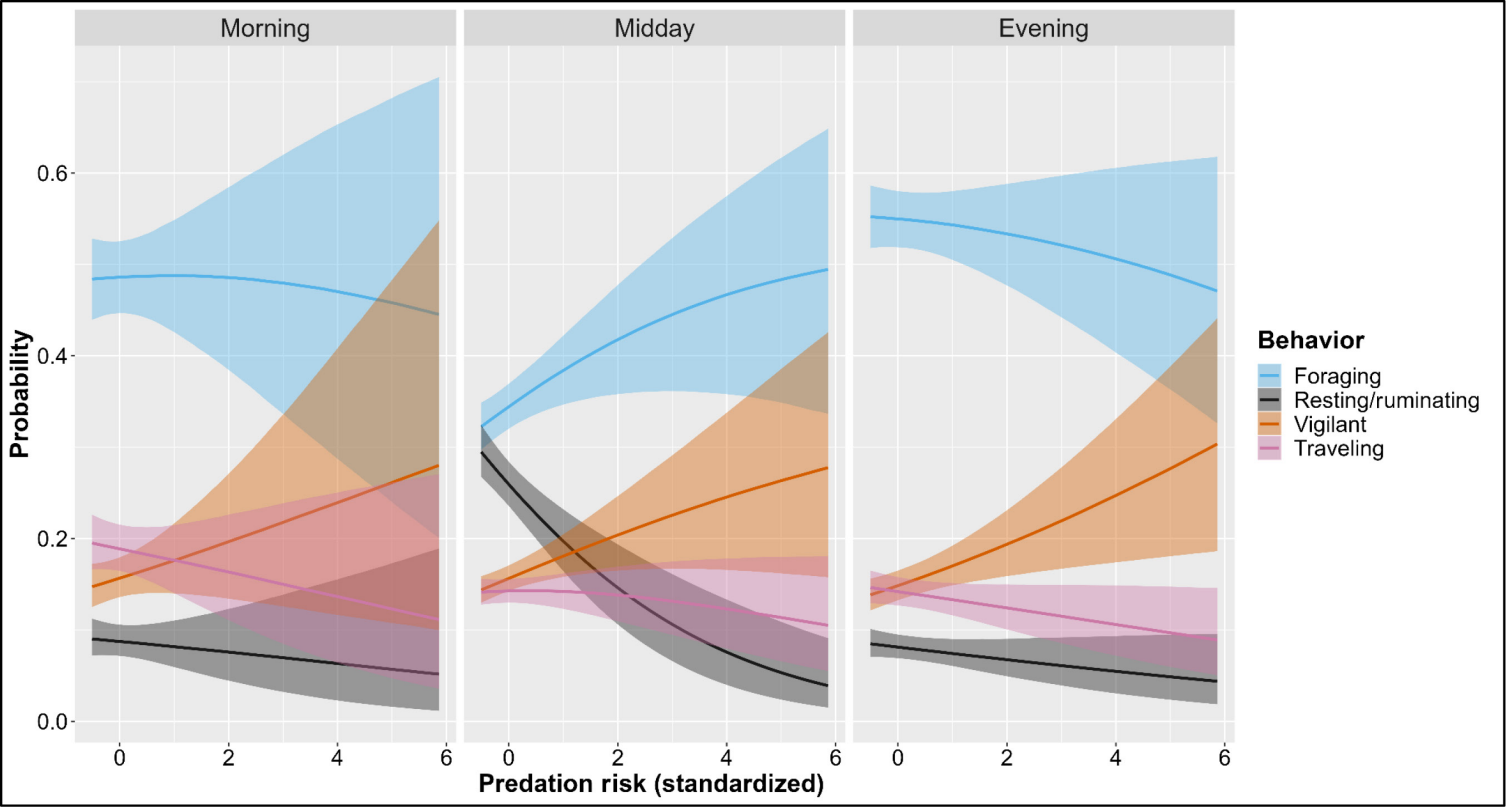
Post hoc influence of diurnal period and Mexican wolf predation risk on adult female elk behavior in west‐central New Mexico and east‐central Arizona 2019–2020. Parameters estimated from the most supported sampling model of focal sampling data. Each panel shows the conditional effect of estimated predation risk by diurnal period on the estimated probability of each behavior with 90% credible intervals. The behavior “other” is removed for clarity.

To investigate if our results differed from previous studies due to the inclusion of midday observations and resting/ruminating behavior, a period and behavior often not included in ungulate behavior analyses, we conducted a post hoc analysis that included the interaction between diurnal period and predation risk in the best‐performing model (Figure 3). Predation risk consistently increased the probability of vigilance during all diurnal periods. During the morning and evening, this increase in vigilance subsequently decreased the probability of foraging. During midday, as predation risk increased, the probability of elk resting/ruminating decreased while foraging increased.

### 5.2 Multitasking

**FIGURE 4:**
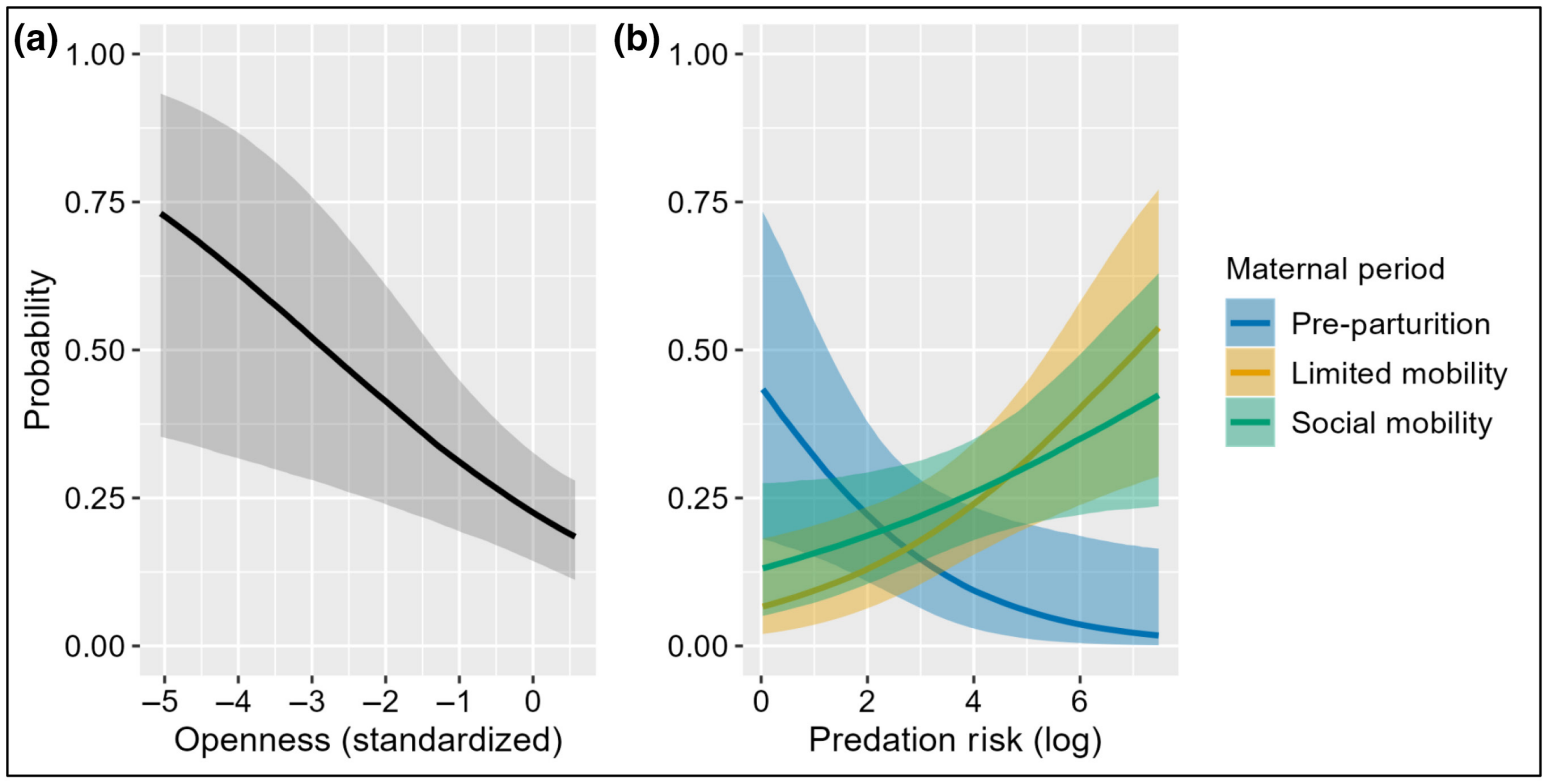
Influence of (a) Openness and (b) Mexican wolf predation risk by maternal period (winter removed for clarity) on adult female elk multitasking behavior in west‐central New Mexico and east‐central Arizona 2019–2020. Parameters estimated from the most supported sampling model of focal sampling data. Each panel shows the conditional effect of a predictor on the estimated probability of multitasking with 90% credible intervals.

To determine the drivers of multitasking we used 1052 occurrences of vigilance in which adult females were recorded as multitasking or not (PPP *n* = 164, LMP *n* = 367, SMP *n* = 421, WP *n* = 100). Whether or not adult female elk multitasked did not depend on the diurnal period. All models were improved by the inclusion of a predation risk metric and its effects were consistent on the probability of multitasking (Table 2). The top performing model for multitasking included openness and an interaction between maternal period and predation risk (risky places relative to wolf use using the PackSY UD at the 2000‐m scale; Table 2). Openness and multitasking had an inverse relationship (−0.75 to −0.14 [90% CrI]; Figure 4a). Increased predation risk had a positive effect on adult female multitasking during the limited/social mobility periods but decreased the probability of multitasking during pre‐parturition (−1.42 to −0.38 [90% CrI]; Figure 4b). Multitasking during winter varied greatly but generally fell between limited/social mobility and pre‐parturition.

**TABLE 2:** Model performance for adult female elk multitasking from focal sampling in west‐central New Mexico and east‐central Arizona 2019–2020.^a^

Model^b^ ^,^ ^c^ ^,^ ^d^	ELPD diff	SE diff	ELPD loo	SE ELPD loo	P loo	SE p loo	Looic	SE looic	PBMA BB Wts
Maternal Period * PackSY2000‐Risky Places^e^ + Openness	0	0	−522.94	16.89	175.59	7.36	1045.88	33.79	0.50
Herd Size + PopSY200‐RSF^e^	−0.73	3.34	−523.66	16.4	173.54	6.95	1047.33	32.8	0.28
Maternal Period + PopSY200‐RSF^e^	−1.37	3	−524.31	16.56	175	7.09	1048.62	33.12	0.15
Null	−4.03	3.53	−526.97	16.17	175.83	6.9	1053.95	32.34	0.04
Diurnal Period * Maternal Period	−5.41	3.78	−528.35	17.32	181.5	7.62	1056.7	34.63	0.03

*Note*: Column definitions: ELPD diff = difference in ELPD value between model and top model. SE diff = difference in standard error of ELPD between model and top model. ELPD loo = ELPD value from approximate leave‐one‐out cross validation. SE ELPD loo = standard error of the ELPD loo value. P loo = effective number of parameters. SE p loo = standard error of p loo value. Looic = −2 * ELPD loo. SE looic = standard error of looic. PBMA BB Wts = pseudo‐Bayesian model averaging with Bayesian bootstrapping weights.

^a^ Models without significant effects removed.

^b^ Within models all main effects of variables in interactions are included but not shown.

^c^ Predation risk indices were created prior to modeling. The first variable indicates the wolf UD used and what scale, in meters.

^d^ All models included a random term for observation.

^e^ Log transformed prior to including in the model.

## 6 DISCUSSION

The observed trade‐offs between foraging, resting/ruminating, and vigilance by adult female elk in response to predation risk from Mexican wolves differed across diurnal periods. By incorporating behaviors (e.g., resting/ruminating) and diurnal periods (e.g., midday) historically not analyzed in predator–prey observational studies, we provide nuance to our understanding of predator–prey dynamics. Multitasking, which may at least partially mitigate the effects of increased vigilance associated with predation risk, depends on maternal period and predation risk. During pre‐parturition, multitasking was negatively associated with predation risk while post‐parturition periods had a positive relationship. By assessing the relationship between Mexican wolf predation risk and multitasking, we provide insight into the ability of prey to make behavioral trade‐offs, depending on their energetic needs, forage resources, and effectiveness of antipredator behaviors, to potentially reduce the detrimental indirect effects of predators on fitness. Predation risk metrics incorporating spatial and temporal components best predicted adult female elk behavior. By developing multiple risk metrics at different spatiotemporal scales, our results provide insight into the complicated predator–prey relationship.

Our study design differed from many previous studies on ungulate behavior because we incorporated essentially all possible behaviors, rather than just vigilance and foraging and we estimated the effects of predation risk on prey behavior across the entire diurnal period. Much of the previous research using behavioral observations either did not report the time of day elk were observed (Fortin et al., 2005; Laundré et al., 2001; Robinson & Merrill, 2013), combined all diurnal periods (Proudman et al., 2020), only observed animals when they were active and easily visible or during peak foraging times (Childress & Lung, 2003; Ciuti et al., 2012; Lung & Childress, 2007; Yiu et al., 2021), or did not provide analysis of prey behavior in relation to the interaction between predation risk and diel periods (Liley & Creel, 2008; Makin et al., 2017; Winnie & Creel, 2007; Wolff & Horn, 2003). Not incorporating all prey behavior or investigating their interaction with the spatiotemporal variation in predation risk (Kohl et al., 2018) may provide an incomplete understanding of trade‐offs prey make due to the NCEs of predators.

We found a positive association between prey vigilance and predation risk across all diurnal periods and an inverse association between vigilance and foraging during crepuscular periods, consistent with previous studies (Childress & Lung, 2003; Laundré et al., 2001; Liley & Creel, 2008; Wolff & Horn, 2003). Increased vigilance is often considered a trade‐off between early predator detection and reduced forage intake (Lima & Dill, 1990, but see Fortin, Boyce, & Merrill, 2004), thus prey should increase vigilance when perceived risk is high and decrease vigilance when risk is low, following the RAH (Lima & Bednekoff, 1999). As predicted, we found evidence of this trade‐off during crepuscular periods when elk are not spatiotemporally avoiding areas of high Mexican wolf predation risk. A few reasons may explain why the trade‐off appeared stronger and had less variability during the evening compared to the morning. First, the sample size for the morning was approximately half that of the evening, likely resulting in wider credible intervals. Second, elk behavior during the night is more variable than during midday, and morning foraging is more centered on sunrise than evening foraging is on sunset (Green & Bear, 1990). This increased variability during the night and our inability to observe elk prior to first light could contribute to the high observed variability in elk behavior during morning as it relates to predation risk. Conversely, elk typically bed midday and begin foraging a few hours prior to sunset (Green & Bear, 1990), allowing for observation of the majority of evening foraging bouts. This difference between behaviors in relation to crepuscular periods may explain why models consisting of combined crepuscular periods did not perform as well as those with crepuscular periods separated.

The reduced foraging and increased vigilances we observed in areas of elevated risk and Mexican wolf activity may be acceptable to elk if risky areas coincide with increased forage availability and/or quality (Brown, 1999). Thompson (2022) reported that, elk generally selected for risky places during crepuscular periods in spring (April 1st–June 30th) and monsoon (July 1st–September 30th) seasons (45% of our data) and that the risky places metric was associated with wet meadows/pastures, aspen stands, low canopy cover, and burns, all of which are preferred foraging areas for elk. Additionally, elk movements often decreased in resource‐rich areas (Frair et al., 2005; Merrill et al., 2020). We observed a negative relationship between traveling and predation risk likely due to elk moving less in preferred foraging areas and more in poorer foraging areas (Hebblewhite & Merrill, 2009; Merrill et al., 2020). These combined results support the RAH by suggesting that reduced foraging time for elk due to increased vigilance in high‐risk areas/times may be a trade‐off elk are willing to make for use of productive foraging areas. Incorporating intake rates, bite size, and forage quantity/quality into future studies will provide more insight into the behavioral, energetic, and fitness trade‐offs made by prey in response to predation risk.

Elk can also reduce the effects of predation risk by taking advantage of low‐risk times, a key component of the RAH (Kohl et al., 2018; Lima & Bednekoff, 1999; Lima & Dill, 1990). Indeed, adult female elk had the highest probability of resting and lowest vigilance in low‐risk areas during times when Mexican wolves are less active during spring and monsoon seasons. By resting/ruminating in low‐risk areas during periods of lower wolf activity, elk can simultaneously meet biological requirements (e.g., sleep; Cirelli & Tononi, 2008) while mitigating predation risk. These results suggest where and when elk rest is potentially constrained by the spatiotemporal variation in predation risk, an understudied aspect of large predator–prey relationships (Lima et al., 2005). When Mexican wolves were less active, high‐risk areas were associated with increased foraging and vigilance by elk at the expense of resting. We were unable to find any studies to directly compare this finding with. However, this relationship is contrary to those in other studies during crepuscular periods. This trade‐off between resting and foraging/vigilance during low‐risk times is typically not incorporated in either theoretical models or empirical data investigating optimal foraging, vigilance, and risk allocation in large ungulates (Fortin, Boyce, & Merrill, 2004; Lima & Dill, 1990; Sih, 1980; but see Hamel & Côté, 2008; Kie, 1999; Seeber et al., 2019).

Thompson (2022) reported that elk more habituated to Mexican wolves (i.e., those occupying areas with higher Mexican wolf use), were more likely to use risky places midday during spring and monsoon than elk with little to no Mexican wolf exposure. These findings fit our results well in that elk observed in high‐risk areas/times had decreased foraging, whereas elk observed in low‐risk areas during riskier times did not (due to their respective vigilance levels). Therefore, our results suggest elk exposed to higher predation risk utilize predicted preferred foraging areas, which appear to be associated with high risk, during midday to make up for lost time foraging during crepuscular periods whereas elk in low‐risk areas do not. These results corroborate the RAH and highlight the ability of prey to alter their behavior to potentially mitigate the NCEs of predators.

Our top model predicted an inverse relationship between herd size and vigilance. These results are consistent with our predictions and theoretical models indicating that larger herds allow for a reduction in individual vigilance and potential costs (e.g., reduced foraging) of detecting predators while simultaneously reducing the probability of death (i.e., detection and dilution hypotheses; Dehn, 1990; Lima & Dill, 1990) for each individual. The advantages of detection and dilution should favor large groups, but an increase in group size also increases intraspecific competition (Fortin, Boyce, & Merrill, 2004; McAlister & Hamilton, 2022) and large herds are often detected, encountered, and attacked more by predators than small herds (Creel & Winnie, 2005; Hebblewhite et al., 2002). The trade‐offs between attacks from predators, intraspecific competition, individual probability of predation, cost of vigilance, forage quality/quantity, multitasking, and herd size are likely deeply intertwined.

As we expected, multitasking was best predicted by an interaction between predation risk and maternal period, with increasing predation risk reducing multitasking during pre‐parturition and increasing it post‐parturition. The energetic demands during pre‐parturition (late gestation, start of lactation, and reduced fat stores from winter) coupled with reduced forage quantity/quality result in a challenging energetic period for adult female elk, which should lead to increased foraging compared to other periods. Additionally, ruminants have a slower passage rate when the fiber content of forage is high, requiring increased search time to maintain daily nutritional requirements and reduce fiber intake (Shrader et al., 2006; Van Soest, 1994, 1996). Our results are consistent with these assumptions showing the highest probability of foraging, the second highest of traveling (behind winter), and the lowest of resting/ruminating during pre‐parturition. Therefore, elk appear to have less spare time available for synchronizing chewing and vigilance due to increased searching time, requiring more intense vigilance in high‐risk areas. Similarly, Yiu et al. (2021) observed more intense vigilance and less multitasking in wildebeests (*Connochaetes taurinus*) and zebras (*Equus quagga*) with higher predation risk, while Sirot et al. (2021) theoretically showed a decrease in multitasking when resources are scarce in high‐risk areas.

Post parturition, during the limited/social mobility periods, as growing days and precipitation increase so does forage quantity/quality. More preferred forage, likely associated with high‐risk areas, requires less search time between bites allowing for an increase in multitasking vigilance. Additionally, less fibrous forage increases ruminating efficiency, which may allow for a decrease in overall foraging time, a pattern shown in our data. This reduction in overall time foraging translated to more time spent resting, potentially providing neonates better access to milk, and vigilance during the limited mobility period when calves are most susceptible to predation. This pattern continued in the social mobility period such that predicted resting is highest, while vigilance and traveling are lowest. These changes manifest via a slight reduction in the probability of multitasking. Unfortunately, our data were insufficient to make a sound assessment of multitasking during the winter, but the activity budgets observed indicate multitasking would fall between those predicted during pre‐parturition and the limited/social mobility periods. Notably, in all maternal periods as predation risk increased, the probability of multitasking and intense vigilance was inversely related, which Yiu et al. (2021) also documented in zebra and wildebeest.

Openness, which is related to both wolf predation risk and forage conditions, was a top predictor of and was inversely related to multitasking. These results were surprising given that our data came primarily from the wet period during our study, which should translate to higher forage availability and therefore, more spare time for multitasking. We suggest a few potential reasons for these results. First, vigilance is more effective in areas with fewer obstructions but head movement, due to chewing, is more detrimental to seeing long distance compared to close distance. Head movement due to chewing may cause elk to prefer intense vigilance in open areas, allowing them to maximize vigilance efficiency. Second, the majority of the multitasking data is from the limited and social mobility periods, therefore it is feasible that predation risk for calves, which is likely greater in areas with less concealment (Barbknecht et al., 2011; Pitman et al., 2014; Shallow et al., 2015), could be related to the increased intense vigilance shown by adult female elk in more open areas. Indeed, our risky places metric indicated that Mexican wolf predation on elk of all ages was more likely in open areas.

Top models in both datasets included a spatiotemporal predation risk metric that included landscape use by Mexican wolves (at the pack level) supporting our prediction that risk indices that incorporate spatial and temporal components of risk should more accurately predict the response of prey to predation risk than those derived from either component alone (Creel et al., 2008; Kohl et al., 2018; Lima & Bednekoff, 1999). The relative intensity of space use by wolves is better accounted for in the pack than the population level UDs, especially when multiple individuals within a pack are collared. By responding to this variation in space use by individual packs, elk may more efficiently navigate the spatial predation risk landscape, potentially allowing them to reduce associated NCEs (Hebblewhite & Merrill, 2007; Horne et al., 2019; Liley & Creel, 2008).

Future research could focus on linking observed behavioral responses (i.e., rates of foraging, resting, vigilance) with fitness metrics such as stress levels, pregnancy rates, nutritional condition, and population dynamics to better disentangle the true extent of NCEs on elk (Prugh et al., 2019). Incorporating the various components of direct predation, such as searching, encountering, and killing would greatly improve our understanding of how elk deal with predation risk by wolves (Creel et al., 2013; Hebblewhite et al., 2005; Lima & Dill, 1990; Suraci et al., 2022). Additionally, routinely incorporating multitasking in future studies on the behavioral plasticity of ungulates will lead to an improved understanding of their ability to offset potential reductions in foraging. To gain a more holistic picture regarding how elk navigate NCEs from predators with different hunting strategies (including humans) and behavioral changes of mothers due to predation risk for calves, future studies could include multi‐predator risk indices (Atwood et al., 2009; Eacker et al., 2016; Makin et al., 2017). Lastly, including all diel periods (i.e., night) and covariates related to individual personalities and nutritional condition could be important for future studies as these time periods/factors likely play an important role in how prey respond to and potentially mitigate the indirect effects of predators (Paterson et al., 2022).

This study furthers our knowledge of predator–prey dynamics, particularly in respect to the effect of spatiotemporal variation in predation risk from a recolonizing predator on prey behavior. Furthermore, we provide novel data on Mexican wolves, a subspecies largely absent from the predator–prey literature, especially in relation to indirect effects on prey. Additionally, we hope to provide ungulate managers with a better understanding of the behavioral plasticity prey may utilize to mitigate the indirect effects of cursorial predators including how prey may alter their behavior as predator recolonization continues. Thus, providing managers a framework for expectations and allowing for more informed decision‐making.

## AUTHOR CONTRIBUTIONS

**Zachary J. Farley:** Conceptualization (lead); data curation (equal); formal analysis (lead); investigation (lead); methodology (lead); writing – original draft (lead); writing – review and editing (lead). **Cara J. Thompson:** Conceptualization (supporting); data curation (equal); methodology (lead); writing – review and editing (equal). **Scott T. Boyle:** Data curation (supporting); investigation (supporting); writing – review and editing (equal). **Nicole M. Tatman:** Conceptualization (equal); funding acquisition (supporting); resources (equal); writing – review and editing (equal). **James W. Cain:** Conceptualization (lead); data curation (equal); formal analysis (equal); funding acquisition (lead); investigation (equal); methodology (lead); project administration (lead); resources (lead); writing – original draft (equal); writing – review and editing (lead).

## CONFLICT OF INTEREST STATEMENT

The authors declare no conflict of interest.

## Figures and Tables

**FIGURE A1:**
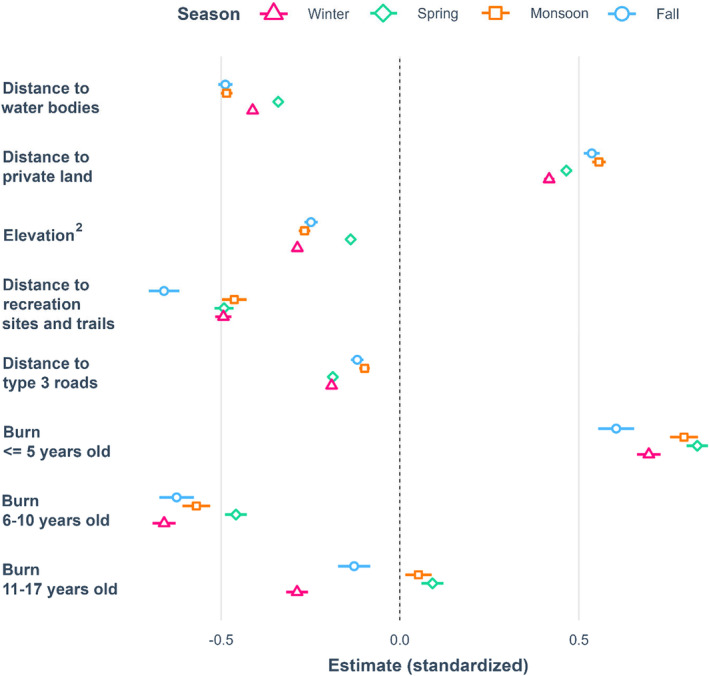
Scaled parameter estimates and 95% confidence intervals from the most supported model for Mexican wolf RSFs by season. Data collected in west‐central New Mexico and east‐central Arizona 2017–2021.

**FIGURE A2:**
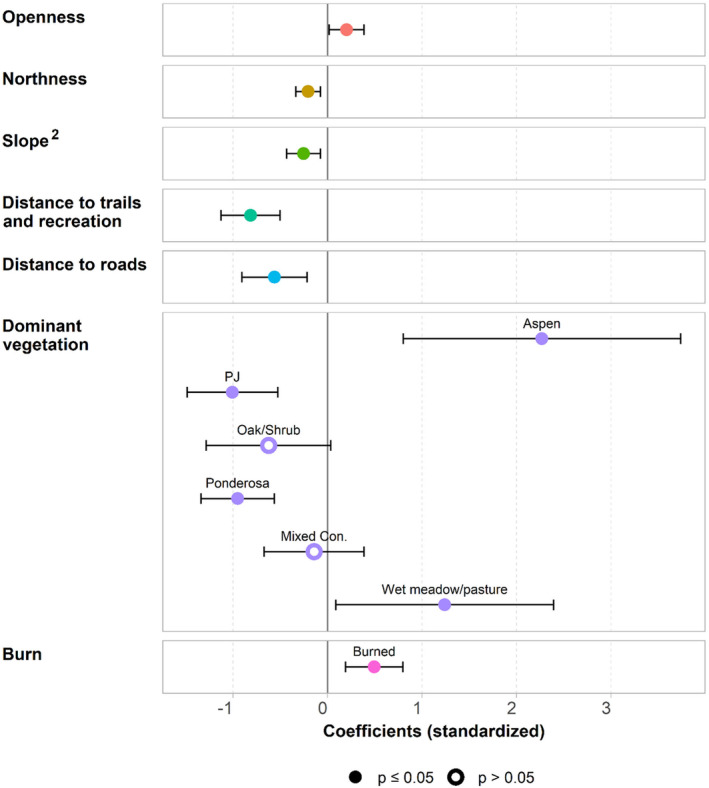
Scaled parameter estimates and 95% confidence intervals from the most supported model for Mexican wolf killed elk (Risky Places). Data collected in west‐central New Mexico and east‐central Arizona 2015–2021.

## Data Availability

Upon publication, the data and corresponding analysis code will be made available at: https://doi.org/10.5061/dryad.ttdz08m48.

## APPENDIX 1

**TABLE A1:** Covariate data source, analysis, and end product.^a^

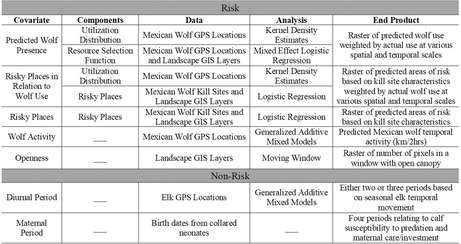

^a^ Covariates from simple GIS layers or observation demographics not included.

## APPENDIX 2 PREDICTED WOLF PRESENCE

### Analysis

We used data from GPS collared Mexican wolves spanning January 2017 to June 2021 to model resource selection functions (RSFs; Thompson, 2022). We defined seasons using biologically (i.e., reproduction; Mech & Boitani, 2003), ecologically (i.e., resource availability; Lafferty et al., 2016; USFWS, 2017), and meteorologically (i.e., weather; see *Study Area*) relevant time frames: spring (April 1st–June 30th), monsoon (July 1st–September 30th), fall (October 1st–November 31st) and winter (December 1st–March 31st). We assigned all locations a season and diel period (Thompson, 2022). We derived four diel periods based on peaks and lulls in wolf activity (km/2 h; Ensing et al., 2014; Thompson, 2022). Collars collected GPS positions every 1, 2, 6 or 13 h depending on various factors (e.g., collar type, time of year, and incorporation in cluster investigations). Most collars collected data at 6 and 13‐h fix rates, therefore, these rates were retained while 1‐and 2‐h fix rates were resampled to 6‐h intervals. Only individuals whose locations occurred in >60% of respective seasons were retained for seasonal RFSs. Approximately 66% of locations from individuals in the same pack were within 380 m of each other. This distance is supported by other studies, which reported inter‐pack distances of 50–1000 m (Benson & Patterson, 2015; Metz et al., 2011; Middleton, Kauffman, McWhirter, Cook, et al., 2013). Therefore, we used this distance as the cut‐off for spatiotemporal independence and as the resolution for our utilization distribution (UD) rasters. When multiple wolves from the same pack were within 380 m of each other at the same point in time, we only used one wolf's location. Additionally, a single location was retained from autocorrelated clusters of points defined as locations within 100 m within 24 h related to captures, food caches, kills, bed sites, and den/rendezvous sites.

Using the average distance traveled by Mexican wolves between 13‐h fixes, we buffered a 100% minimum convex polygon (MCP) derived from all remaining wolf locations and excluded bodies of water rarely used by wolves (i.e., lakes; Thompson, 2022). Within the MCP we generated, avoiding duplication, random locations with used locations at a 10:1 ratio. We extracted values for openness, fire age, burn severity, elevation, canopy cover, slope, vector ruggedness measure (VRM), aspect, terrain ruggedness index (TRI), horizontal cover, snow depth, road density, dominant vegetation, human density, distance to roads, water (water bodies, artificial water points, and combined perennial water features), recreation areas, private lands, trails, and ecotone via GIS layers in Program R for both used and random locations (Table A2). To estimate the pack‐level UDs, all non‐dispersing and spatiotemporal independent GPS points within a pack were used and weighted by pack size.

We applied a linear transformation by adding one to all RSF values and two to all UD values prior to calculating the product of UD and RSF to create the predicted wolf presence metric. We used this transformation for three reasons. First, it prevents observations from ever having a value of zero due to only one metric (UD or RSF) being a zero. Second, in situations when both UD and RSF values are <1, the product would be less than either the UD or RSF. This reduction is contrary to our belief of how elk perceive risk, which is that a greater response is expected in areas of both documented use (i.e., UD >0) and predicted selection, compared to areas with only either use or predicted selection (Hebblewhite et al., 2005). Lastly, from an ecological perspective, UDs should be more strongly related to elk behavior than RSFs as UDs represent actual use by wolves and RSFs represent potential use (Hebblewhite & Merrill, 2007). To avoid collinearity, predictors with a Spearman's rank correlation coefficient of ≥0.7 were not modeled together. Additionally, we tested variables for near zero variance and standardized, when applicable, seasonally. Using mixed effects logistic regression with pack as a random term, Thompson (2022) compared either 40 or 45 *a priori* models (Table A3), depending on season, via AIC_c_ and assessed model fit of the most supported model(s) within season with 10‐fold cross‐validation run 100 times in Program R. Model variance inflation factors (VIF) was tested to insure independence between model terms (i.e., VIF <2).

### Results

Mexican wolf selection was modeled using 137 individuals from 58 packs. Each season had the same single top model structure (Table A4). The most influential variable across all seasons on Mexican wolf resource selection was fire age, followed by elevation, then distances to trails/recreation areas, private land, water bodies, and type 3 roads (Figure A1). Only relative selection for fire age and distance from trails/recreation areas differed across seasons. Fire age was the strongest landscape characteristic estimating selection. Mexican wolves avoided 6–10 year‐old burns and selected younger burns (<5 years) more than unburned areas. Seasonal burn selection differed such that Mexican wolves avoided older burns (11–17 years) in fall and winter but selected for them in spring and monsoon seasons. Mexican wolves selected for moderate elevation, shorter distances to water bodies, type 3 dirt roads (high clearance vehicle/4WD), and trails/recreation areas while avoiding private land. Average rho values from 10‐fold cross‐validation on top seasonal models ranged from 0.991 to 0.998, indicating consistent and strong predictive accuracy.

**TABLE A2:** Landscape covariates, their source, and manipulation used in RSF (*) and RP (♦) predation risk metrics for Mexican wolves.

Variables used in modeling	Source	Data manipulation
Dominant vegetation type*♦	LANDFIRE	Reclassified into 8 categories. Grassland, aspen, other, oak/shrub, piñon‐juniper, ponderosa, mixed conifer, wet meadow/pasture
Slope*♦	LANDFIRE	None
Terrain ruggedness index*♦	–	Created in R using spatialEco package and DEM
Vector ruggedness metric*♦	–	Created in R using spatialEco package and DEM
Distance to water*♦	USGS, USFS, BLM	Combined natural perennial water bodies and artificial water sources together
Distance from road (4 types)*♦	USFS, BLM, AZDOT	Merged road layers and reclassified into 4 categories based on use: (1) paved/well‐maintained dirt roads with a relatively high degree of travel, accessible with low clearance vehicles, (2) gravel and dirt roads which experience less travel and/or require moderate clearance, (3) unmaintained dirt roads requiring a high clearance vehicle, (4) all roads
Road density (4 types)*♦	–	Created density rasters for each road class by quantifying km of roads in each 1.5 × 1.5 km area
Distance from ecotone*♦	–	Reclassified dominant vegetation layer as treed or open and defined an ecotone of where they met. I extracted ecotone as a line vector and buffered each side by 50 m
Distance to private land*♦	BLM	Extracted private and local government land polygons from surface ownership map to create a private lands polygon
Human density*	CIESIN NASA SEDAC	None
Years since fire*♦	MTBS, NIFC	Calculated time since most recent fire using GPS timestamp. Reclassified into 4 categories: unburned or fires >17 years in age, 0.1–5, 6–13, and 14–17 years. Fires after a point was taken were excluded.
Fire burn severity*♦	MTBS	Reclassified into 4 categories: unburned or masked pixels, low severity or improved greenness, moderate severity, high severity. Using YSF, I reclassified areas burned more than 17 years ago as unburned. I selected the most severe burn value experienced in the years 2000–2021 for a location and excluded any from fires after the point was taken. I converted the reclassified values as continuous (Crabb et al., 2022)
Burned area*♦	–	Created a binary variable using YSF and BS_Max_ values: unburned and burn
Elevation*♦	LANDFIRE	None
Snow depth*	NOAA NOHRSC	None
Distance to fence♦	USFS, BLM	None; not available for tribal lands
Percent canopy cover*♦	MRLC	None
Horizontal cover*♦	LANDFIRE	Created using reclassified existing vegetation height and cover data. I condensed vegetation height to <1 m (open), 1 to <2 m (tall enough to hide a wolf or lion), >2 m (fully obstructed to an elk). I condensed vegetation cover into open (0%–30%) and closed (>30%). I then multiplied these values together
Distance to recreational areas and trails*♦	USFS, BLM	Digitized polygons for ski resort, Sipe wildlife refuge viewing area and visitor's center, and extremely popular fishing/boating areas and combined with campground locations. Combined hiking trails, OHV trails, and recreational sites together
Openness*♦	–	Reclassified canopy cover into binary layer of <30% canopy cover and ≥30% canopy cover. Summed number of open pixels within a focal pixel centered within a 250 × 250 m or 500 × 500 m rolling window

**TABLE A3:** The structures of a priori models used to evaluate seasonal Mexican wolf resource selection from GPS and GIS data in west‐central New Mexico and east‐central Arizona 2017–2021.

Model no.	Model structure^a^ ^,^ ^b^ ^,^ ^c^ ^,^ ^d^
1	Private + Elevation^2^ + Rec/trails + Water + Road 3 + YSF
2	Private + Elevation^2^ + Rec/trails + Water + Road 3 Density + YSF
3	Private + Elevation^2^ + Rec/trails + Water + Road 2 Density + YSF
4	Private + Elevation^2^ + Rec/trails + Water + YSF
5	Private + Elevation^2^ + Slope + Water + YSF
6	Private + Elevation^2^ + Road 3 + Water + YSF
7	Private + Elevation^2^ + YSF + Water + MBS * YSF
8	Private + Elevation^2^ + Rec/trails + Water + Burn * Diel Period
9	Private + Elevation^2^ + Rec/trails + Water + Burn
10	Private + Elevation^2^ + Rec/trails + YSF + Slope
11	Elevation^2^ + Rec/trails + Water + Road 3 + YSF
12	HD + Elevation^2^ + Rec/trails + Water + YSF
13	Rec/trails + Elevation^2^ + YSF + Water + MBS * YSF
14	Private + Road 3 + Rec/trails + Water + Burn
15	Private + Elevation^2^ + Road 3 + Slope + YSF
16	Private + Elevation^2^ + Openness + Water + Rec/trails
17	Private + Elevation^2^ + Canopy Cover + Water + Rec/trails
18	Private + Elevation^2^ + Rec/trails + Road 3 + YSF
19	Private + Openness^2^ + Water + Rec/trails
20	Private + Canopy Cover^2^ + Water + Rec/trails
21	Private + Elevation^2^ + Water + Road 3 + Rec/trails
22	Private + Elevation^2^ + Water + Rec/trails + Slope
23	Private + Elevation^2^ + Water + Rec/trails + Diel Period * Elevation
24	Private + Elevation^2^ + Water + Road 2 Density + Rec/trails
25	Private + Veg + Rec/trails + Water + MBS * YSF
26	Private + Elevation^2^ + Water + Openness * Elevation
27	Private + Elevation^2^ + Water + Canopy Cover * Elevation
28	Private + Water + Canopy Cover * Elevation^2^
29	Private + Elevation^2^ + Canopy Cover^2^ + Rec/trails
30	Private + Elevation^2^ + Openness^2^ + Rec/trails
31	Private + Elevation^2^ + Water + Road 3 + Slope
32	Elevation^2^ + Water + Diel Period * Private
33	Private + Elevation^2^ + Water + Diel Period * Elevation
34	Private + Elevation^2^ + Diel Period * Rec/trails
35	Rec/trails + Elevation^2^ + Water + Road 3 + HD
36	HD + Elevation^2^ + Water + Diel Period * HD
37	Water + Elevation^2^ + Diel Period * Openness
38	Private + Veg + Water + MBS * YSF
39	Private + Elevation^2^ + Diel Period * Openness
40	Veg + Rec/trails + Water + Diel Period * Private
41	Private + Elevation * Snow Depth + Road 3 + YSF^e^
42	Private + Elevation * Snow Depth + Road 3 + Road 2^e^
43	Private + Elevation^2^ + Snow Depth + Openness + Road 2 + YSF^e^
44	Private + Elevation^2^ + Slope + Snow Depth + Road 3^e^
45	Private + Slope + Rec/Trails + Snow Depth + Road 3 + Burn^e^

^a^ Variable notation: Private = distance to private land. Rec/trails = distance to nearest recreation area or trail. Water = distance to nearest water body. Road 2 = distance to level 2 road. Road 3 = distance to level 3 road. MBS = max burn severity, 4 categories: *unburned, low severity, moderate severity, and high severity. YSF = years since fire, 4 categories: *unburned or >17 years old, >0 and ≤5 years old, >5 and ≤10 years old, and >10 and ≤17 years old. HD = human density. Veg = vegetation community, 8 categories: *grassland, oak/shrubland, aspen, pinon‐juniper, ponderosa, mixed conifer, wet meadow/pasture, other. *Reference category.

^b^ Within models all main effects of variables in interactions are included but not shown.

^c^ Within models all main effects of squared variables are included but not shown.

^d^ All models included a random term for pack.

^e^ Only used for winter data.

**TABLE A4:** Comparison of the top three models of seasonal Mexican wolf resource selection functions with data collected from west‐central New Mexico and east‐central Arizona 2017–2021.

Model^a^ ^,^ ^b^ ^,^ ^c^	*K*	AIC_c_	ΔAIC_c_	ModelLik	AIC_c_Wt	LL
Spring						
Private + Elevation^2^ + Rec/trails + Water + Road 3 + YSF	11	200991.77	0.00	1.00	1.00	−100484.88
Private + Elevation^2^ + Rec/trails + Water + Road 3 Density + YSF	11	201600.81	609.04	0.00	0.00	−100789.40
Private + Elevation^2^ + Rec/trails + Water + Road 2 Density + YSF	11	201881.00	889.32	0.00	0.00	−100929.54
Monsoon						
Private + Elevation^2^ + Rec/trails + Water + Road 3 + YSF	11	137302.48	0.00	1.00	1.00	−68640.24
Private + Elevation^2^ + Rec/trails + Water + Road 3 Density + YSF	11	137366.01	63.54	0.00	0.00	−68672.01
Private + Elevation^2^ + Rec/trails + Water + Road 2 Density + YSF	11	137430.66	128.19	0.00	0.00	−68704.33
Winter						
Private + Elevation^2^ + Rec/trails + Water + Road 3 + YSF	11	246639.75	0.00	1.00	1.00	−123308.87
Private + Elevation^2^ + Rec/trails + Water + Road 3 Density + YSF	11	247233.89	594.14	0.00	0.00	−123605.95
Private + Elevation^2^ + Rec/trails + Water + Road 2 Density + YSF	11	247798.10	1158.35	0.00	0.00	−123888.05

*Note*: The number of parameters (*K*), Akaike's Information Criterion adjusted for small sample size (AIC_c_), ΔAIC_c_, relative likelihood of the model given the data (ModelLik), Akaike weights, and log‐likelihood presented.

^a^ Variable notation: Private = distance to private land. Rec/trails = distance to closest recreation area or trail. Water = distance to water body. Road 3 = distance to level 3 roads. YSF = years since fire, 4 categories: *unburned or >17 years old, >0 and ≤5 years old, >5 and ≤10 years old, and >10 and ≤17 years old. *Reference category.

^b^ Models including a squared term, also included its main effect.

^c^ All models included a random term for pack.

## APPENDIX 3 MEXICAN WOLF RISKY PLACES MODEL DEVELOPMENT, COMPARISON, AND VALIDATION

### Analysis

Thompson (2022) used female elk and calves (<6 months) determined to have been killed by Mexican wolves from mortality investigations to estimate risky places. Mortality investigations consisted of assigning cause‐specific mortality during necropsies, based on identifying wounds, subcutaneous hemorrhaging, canine spacing/diameter, track/scat/hair identification, distinct consumption patterns, signs of struggle/chase, and blood trails (Alt & Eckert, 2017; Kautz et al., 2019; Verzuh et al., 2018). We combined 95% kernel density estimated wolf territory polygons containing these kill sites to represent the available area for the risky places analysis. A 10:1 ratio of random points to kill locations was generated across the merged polygons with a minimum distance of 400 meters between random points. Random and kill site locations were buffered by 200 meters to account for the way in which wolves hunt (i.e., cursorily; Mech & Boitani, 2003). Kill‐sites not occurring in an established wolf territory were assigned to the nearest pack and packs containing a single predation event (*n* = 3) were combined with a spatially overlapping territory containing more kill‐site data.

Covariate values extracted from GIS layers for kill‐sites and random locations include: road density, dominant vegetation community, elevation, fire age, slope, burn severity, aspect, openness, horizontal cover, VRM, TRI, as well as distance to ecotone, fence, roads, water, private land, and trails/recreation areas (Appendix 2: Table A2). We used the mean value for continuous predictors and majority value for categorical predictors. Locations classified as dominant vegetation community “other” occurred infrequently enough to not be represented in both kill and random sites, causing very large standard errors and issues with parameter estimation and thus were combined with grasslands after cross‐referenced satellite imagery revealed the vegetation to be functionally similar. Burn severity and fire age were used to create a binary burn category and aspect was transformed to create a northness metric.

We ran models in Program R using mixed effects logistic regression with a random term for wolf pack (Thompson, 2022). We then compared models via AIC_c_. We validated the most supported models with 10‐fold cross validation run 100 times and tested variance inflation factors (VIFs) to ensure independence between model terms (i.e., <2). Seasonal variation in risky places was not evaluated due to small sample sizes of multiple packs with multiple kill‐sites within seasons.

### Results

From February 2015 through September 2021, 392 adult female elk and calves (<6 months) were determined to be killed by 29 packs. Elk <6 months old comprised the majority of kills (*n* = 226; 58%), whereas adult females and yearlings occurred similarly (*n* = 89; 23% and *n* = 77; 20% respectively). Three models were within ΔAIC_c_ ≤2 of the model with the lowest AIC_c_ (Table A5). Models with uninformative parameters were removed, resulting in a single top model.

The probability of a location being an elk kill was influenced by dominant vegetation community, distance to trails/recreation areas, distance to roads, burn history, slope, and openness at 500 m. Aspen and wet meadows/pasture had a significantly higher likelihood of containing kills compared to grassland while both piñon‐juniper and ponderosa forests had a significantly lower likelihood of containing kills than grassland. Mixed conifer forests and oak shrubland did not differ in their likelihood of kills compared to grasslands at the 95% confidence level. The likelihood of an elk being killed by Mexican wolves was higher closer to all road types, closer to trails and recreation areas, in burns, in more open areas, on low to moderate slope angles, and in south facing terrain (Figure A2). The average rho value of 10‐fold cross‐validation accounting for mixed effects was 0.88 ± 0.067 (SD), indicating good predictive accuracy.

**TABLE A5:** Comparison of models with ΔAIC_c_ <5 for Mexican wolf risky places models in west‐central New Mexico and east‐central Arizona 2015–2021.

Model^a^ ^,^ ^b^ ^,^ ^c^ ^,^ ^d^	*K*	AIC_c_	∆AIC_c_	ModelLik	AIC_c_Wt	LL	Cum.Wt
Openness + Rec/trails + Veg + Northness + Slope^2^ + Any Road + Burn	15	1827.39	0	1	0.36	−898.64	0.36
Openness + Rec/trails + Water + Private + Veg + Northness + Slope^2^ + Burn + Any Road	15	1830.16	2.76	0.25	0.09	−900.02	0.45
Openness + Rec/trails + Veg + Northness + Slope^2^ + Any Road + YSF	17	1830.35	2.95	0.23	0.08	−898.10	0.53
Rec/trails + Water + Private + Veg + Northness + Slope^2^ + Any Road + Burn	16	1830.87	3.47	0.18	0.06	−899.37	0.59
Rec/trails + Private + Veg + Northness + Slope^2^ + Any Road + Burn	15	1830.92	3.53	0.17	0.06	−900.40	0.65
Openness + Rec/trails + Water + Veg + Northness + Slope^2^ + Any Road + YSF	18	1830.94	3.55	0.17	0.06	−897.39	0.71
Horizontal Cover + Rec/trails + Private + Veg + Northness + Slope^2^ + Any Road + Burn	16	1831.07	3.67	0.16	0.06	−899.47	0.77
Horizontal Cover + Rec/trails + Private + Veg + Northness + Slope^2^ + Any Road + Burn + Water	17	1831.1	3.7	0.16	0.06	−898.48	0.83
Canopy Cover + Rec/trails + Water + Private + Veg + Northness + Slope^2^ + Burn + Any Road	17	1831.42	4.03	0.13	0.05	−898.64	0.88
Rec/trails + Water + Private + Veg + Northness + Slope^2^ + Any Road + Openness + YSF	19	1831.98	4.59	0.1	0.04	−896.9	0.92

*Note*: The number of parameters (*K*), Akaike's Information Criterion adjusted for small sample size (AIC_c_), ΔAIC_c_, relative likelihood of the model given the data (ModelLik), Akaike weights, log‐likelihood, and cumulative Akaike weights presented.

^a^ Variable notation: Rec/trails = distance to nearest recreation area or trail. Water = distance to nearest water body. Any Road = distance to any road. YSF = years since fire, 4 categories: *unburned or >17 years old, >0 and ≤5 years old, >5 and ≤10 years old, and >10 and ≤17 years old. Veg = vegetation community, 8 categories: *grassland, oak/shrubland, aspen piñon‐juniper, ponderosa, mixed conifer, wet meadow/pasture, other. Burn = 2 categories, *unburned or >17 years old, and burned. Private = distance to nearest private land. *Reference category.

^b^ Within models all main effects of variables in interactions are included but not shown.

^c^ Within models all main effects of squared variables are included but not shown.

^d^ All models included a random term for pack.

## APPENDIX 4

**TABLE A6:** Literature summary of variables shown to influence elk behavior and justification for utilization in a priori models analyzing adult female elk behavior in west‐central New Mexico and east‐central Arizona 2019–2020.

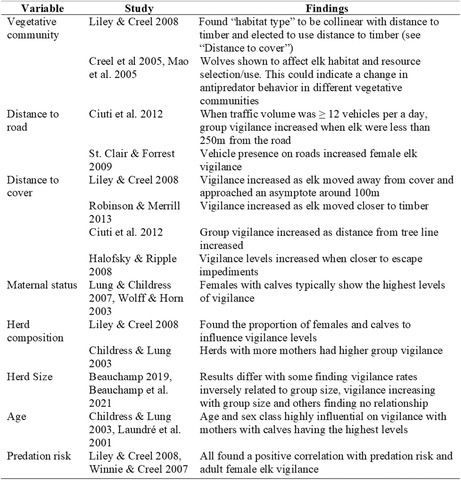

**TABLE A7:** The structures of a priori models used to evaluate adult female elk behavior from focal sampling in west‐central New Mexico and east‐central Arizona 2019–2020.

Model no.	Model structure^a^ ^,^ ^b^
1	Diurnal Period * Herd Size
2	Diurnal Period + Proportion Cows
3	Diurnal Period * Predation Risk
4	Diurnal Period + Herd Size + Predation Risk
5	Diurnal Period + Herd Size * Predation Risk
6	Diurnal Period + Proportion Cows + Predation Risk
7	Diurnal Period + Proportion Cows * Predation Risk
8	Diurnal Period + Distance to Cover * Predation Risk
9	Diurnal Period + Maternal Period * Predation Risk + Distance to Cover * Predation Risk
10	Diurnal Period + Maternal Period + Distance to Cover + Predation Risk
11	Diurnal Period + Herd Size + Proportion Cows + Slope + Predation Risk

^a^ Within models all main effects of variables in interactions are included but not shown.

^b^ All models contain a random term for observation.

**TABLE A8:** The structures of a priori models used to evaluate adult female elk multitasking from focal sampling in west‐central New Mexico and east‐central Arizona 2019–2020.

Model no.	Model structure^a^ ^,^ ^b^
1	Herd Size + Maternal Period
2	Herd Sie * Maternal Period
3	Maternal Period + Slope
4	Diurnal Period * Maternal Period
5	Diurnal Period * Slope
6	Cover * Predation Risk
7	Predation Risk + Herd Size
8	Predation Risk + Maternal Period
9	Predation Risk * Maternal Period

^a^ Within models all main effects of variables in interactions are included but not shown.

^b^ All models contain a random term for observation.
